# The structural basis for synergistic inhibition of geranylgeranyl diphosphate synthase with stereoisomeric triazole bisphosphonates

**DOI:** 10.1063/4.0001175

**Published:** 2025-10-27

**Authors:** Andrew Pham, Sarah Holstein, Gloria Borgstahl

**Affiliations:** UNMC, Omaha, NE, USA

## Abstract

Several incurable cancers are characterized by abnormal protein production and secretion. A prime example being multiple myeloma and its excessive production of non-functional antibodies (M protein). Proper secretion of M protein is necessary for cancer cell survival but is mediated by intracellular trafficking events. These trafficking events are dependent on the Rab family of proteins which require a post-translational geranylgeranylation for effective localization to the membrane. Through disruption of Rab geranylgeranylation and consequently, abnormal protein secretion, M protein excessively builds up within the myeloma cell inducing the unfolded protein response and apoptosis. An effective strategy to disrupt Rab geranylgeranylation is to inhibit geranylgeranyl diphosphate synthase (GGDPS) which synthesizes the post-translationally added prenyl group.

Our collaborators have synthesized a pair of stereoisomeric triazole bisphosphonates with high selectivity for GGDPS. These drugs have been named the homoneryl (HN: Z isomer) and homogeranyl (HG: E isomer) compounds. Interestingly, these become more potent GGDPS inhibitors when used as a 3:1 mixture (HG:HN), suggesting synergism. Our goal is to solve the structures of each isomer individually bound to GGDPS as well as together to understand the role of stereochemistry in structure-guided drug development.

In our attempts to co-crystallize drug-bound structures of GGDPS, we instead crystallized a product-bound variant and solved its structure to the highest resolution yet for human GGDPS. Given the difficulty in crystallizing human GGDPS, we instead switched to yeast GGDPS for our drug co-crystals. Crystal structures of yeast GGDPS with each drug individually and as a combination revealed that each stereoisomer binds the same substrate pocket.

Structures with samples treated with both drugs still showed density for the same single substrate binding pocket. Drug treated human GGDPS cryo-EM structures revealed a similar finding, with individual treatment of each stereoisomer showing single drug occupancy in the same substrate pocket. Although the general binding pocket is shared between the two stereoisomers, restraining the drugs to either an E or Z isomer for ligand fitting reveals a 180-degree flip of the triazole ring group. Additionally, the overall quaternary arrangement differs between the HG and HN treated GGDPS structures. While the hexameric structure of GGDPS is overall conserved, the association between one of the dimers is oriented differently, more so in the HG structure than the HN.

